# Exploring the causal relationship between inflammatory cytokines and migraine: a bidirectional, two-sample Mendelian randomization study

**DOI:** 10.1038/s41598-023-46797-3

**Published:** 2023-11-08

**Authors:** Chong Fu, Yan Chen, Wei Xu, Yanping Zhang

**Affiliations:** Department of Gastroenterology, Anqing Municipal Hospital, 352#, Renmin Road, Anqing, Anhui 246000 People’s Republic of China

**Keywords:** Medical research, Risk factors

## Abstract

To further evaluate the causal relationships between inflammatory cytokines and migraine, we conducted a bidirectional, two-sample Mendelian randomization (MR) analysis using genetic data from publicly available genome-wide association studies (GWAS). We used several MR methods, including random-effect inverse-variance weighting (IVW), weighted median, MR-Egger, to test the causal relationships. Sensitivity analyses were also conducted to evaluate the robustness of the results. The results showed that hepatocyte growth factor (HGF) was positively associated with the risk of migraine (odds ratio [OR], 1.004; 95% confidence interval [CI], 1.001–1.008; P = 0.022). In addition, Interleukin-2 (IL-2) was considered a downstream consequence of migraine (OR, 0.012; 95% CI, 0.000–0.0929; P = 0.046). These findings suggest that HGF may be a factor associated with the etiology of migraine, while IL-2 is more likely to be involved in the downstream development of migraine.

## Introduction

Migraine is a recurrent, chronic neurological disorder that can be disabling. It is characterized by episodic throbbing headaches, often accompanied by nausea and/or vomiting, and in some cases, photophobia, phonophobia, anxiety, and depression^1^. In 2019, migraine ranked second among single neurological diseases in terms of burden, and is listed as the seventh leading cause of disability globally by the World Health Organization, and the leading cause of disability in young people under the age of 50^2,3^.

While migraine has traditionally been categorized as a neurological disorder, its underlying pathophysiological mechanisms remain enigmatic. A growing body of evidence indicates that it may encompass a state of “aseptic inflammation” localized within the intracranial meninges, often referred to as neurogenic inflammation. Neurogenic inflammation is a type of inflammation that is associated with the nervous system. It is characterized by the production of inflammatory mediators, increased vascular permeability, leukocyte infiltration, and damage to the blood–brain barrier and activation of glial cells. When the trigeminal ganglion and its fibers are stimulated, they release large amounts of neuropeptides, such as calcitonin gene-related peptide (CGRP). CGRP, on the one hand, causes vasodilation, which has a “pro-inflammatory effect.” On the other hand, it binds to receptors on the dura mater, causing mast cell degranulation and the release of various inflammatory factors, leading to aseptic inflammation. This inflammation can then lead to overexcitation of neurons, which in turn sensitizes pain receptors in the meninges, ultimately leading to a migraine attack^4,5^. Epidemiological investigations have documented the co-occurrence of migraine alongside various chronic inflammatory conditions, including multiple sclerosis, chronic inflammatory rheumatic diseases, and inflammatory bowel disease^6–8^. Furthermore, systemic factors such as cytokines and the gut microbiome have the potential to exacerbate or enhance the susceptibility to migraine^9^. Expanding upon the framework of neurogenic inflammation, multiple studies have delved into the intricate relationship between headache disorders and assorted inflammatory markers. In individuals suffering from migraine, pro-inflammatory cytokines such as Interleukin 1 beta (IL-1β), Interleukin 6 (IL-6), and Tumor necrosis factor alpha (TNF-α), along with anti-inflammatory cytokines like Interleukin 10 (IL-10), are postulated to exert an influential role in the pathogenesis of migraine^10^. The scrutiny of numerous peripheral inflammatory biomarkers, including the neutrophil-to-lymphocyte ratio (NLR) and the neutrophil-to-monocyte ratio (NMR), across a spectrum of headache disorders has offered insights suggesting a potential connection between systemic inflammatory processes and the pathophysiology of migraine^11^. Nevertheless, it remains presently uncertain whether these inflammatory markers are causally intertwined with the pathophysiology of migraine or if they possess a causal relationship with the condition.

MR stands as a robust tool in the realm of epidemiological research, predicated on its fundamental principle of employing genetic variations as instrumental variables to appraise the causal associations between risk factors and specific diseases^12,13^. Epidemiological studies grapple with the substantive challenge of confounding factors, which impede the delineation of causal inferences. In the context of MR, genetic variations conform to the precepts of allele randomization to offspring, mirroring the design of a randomized controlled trial^14,15^. The MR methodology adeptly surmounts impediments such as confounding variables, reverse causality, and issues of representativeness in observational studies. Additionally, it addresses the practical feasibility concerns that often beset randomized controlled trials.

Nonetheless, it is noteworthy that, hitherto, no investigation has harnessed the potential of MR to scrutinize the nexus between inflammatory cytokines and migraine. Consequently, this study endeavors to employ the MR framework, using two-sample methodologies, to elucidate the plausible causal link between inflammatory cytokines and migraine. The overarching objective is to furnish a theoretical underpinning for comprehending the intricate interplay between inflammatory cytokines and the genesis and progression of migraine.

## Methods

### MR assumptions

In this investigation, we conducted a comprehensive analysis by aggregating data from a genome-wide association study dataset, employing a two-sample MR approach to explore the bidirectional links between inflammatory cytokines and the incidence of migraines. Specifically, the forward MR analysis scrutinized the role of inflammatory cytokines as the exposure and their impact on migraine as the outcome, while the reverse MR analysis delved into migraine as the exposure and its potential influence on inflammatory cytokines as the outcome. Furthermore, we performed sensitivity analyses to assess the robustness of our findings. It is crucial to highlight that MR studies hinge on the satisfaction of three fundamental assumptions: (1) the instrumental variables must demonstrate a strong association with the exposure factor; (2) the instrumental variables should exhibit no correlation with any confounding factors pertaining to the “exposure-outcome” relationship; (3) The instrumental variables can solely influence the outcome variable through the exposure factor.

### Data source

In this Mendelian Randomization analysis, we employed two datasets, both derived from publicly accessible GWAS data. The dataset pertaining to inflammatory cytokines was sourced from a comprehensive study, providing genetic variation information for 41 distinct inflammatory cytokines among a cohort of 8293 individuals of Finnish descent. This investigation harmonized data from the Finnish Young Finns Study (YFS) and the FINRISK survey, revealing an average participant age of 37 years for the YFS study and 60 years for the FINRISK survey^16^.The migraine-related dataset predominantly originated from the UK Biobank (https://gwas.mrcieu.ac.uk/) via the Mr-Base platform. This dataset encompasses a meta-analysis study incorporating 13,597 cases of European ancestry afflicted by migraines and 449,336 control subjects, participants were of European ancestry and included both men and women. The summary statistics covered all migraine cases, regardless of subtype. UK Biobank, the largest resource of genetic and environmental factors associated with disease causation or prevention in the UK, enrolled participants aged 40–60 years.

### Instrumental variable selection

In the context of forward MR analysis, we applied a rigorous significance threshold (P < 5 × 10^−8^), signifying genome-wide significance, to select Single Nucleotide Polymorphisms (SNPs) strongly associated with inflammatory cytokines. However, due to limited SNP identification when specific inflammatory cytokines were considered as exposure factors, we adopted a more stringent threshold (P < 5 × 10^−6^). Additionally, we implemented SNP clustering (with a distance threshold of 10,000 kilobases and an r^2^ value of 0.001) to address issues stemming from linkage disequilibrium.In contrast, for the reverse MR analysis, we identified SNPs significantly associated with migraines at the genome-wide level (P < 5 × 10^−8^) and characterized by low linkage disequilibrium (r^2^ < 0.001, distance threshold = 10,000 kilobases). These specific SNPs were chosen to investigate the causal impact of migraines on inflammatory cytokines. To evaluate the potential presence of weak instrument bias, we calculated the F-statistic, with an F-value exceeding 10 indicating the absence of such bias, thus further substantiating the causal assumptions. The formula for computing the F-statistic is F = [(N−K−1)/K] × [R^2^/(1−R^2^)], where N represents the sample size for the exposure factor, K denotes the number of instrumental variables, and R^2^ signifies the proportion of exposure factor variance explained by these instrumental variables.

### Statistical analysis

We utilized five distinct MR methods to investigate the causal association between inflammatory cytokines and migraines. The primary MR analysis method employed was the IVW approach, complemented by MR Egger, Weighted Median, and other supplementary methods for further analysis. Significance was attributed to a causal effect when the P-value was less than 0.05, denoting an increased risk of the outcome attributable to the exposure. The potential causal relationship between inflammatory cytokines and migraines was assessed using odds ratios (OR).To assess heterogeneity, Cochran’s Q statistic was applied in both the MR Egger and IVW methods, and heterogeneity was considered absent when the p-value exceeded 0.05. In instances of heterogeneity, random-effects models were employed to either exclude or estimate SNP effects. We examined pleiotropy using the intercept p-value derived from MR Egger regression, with a p-value exceeding 0.05 indicating the absence of potential pleiotropic effects. Furthermore, we conducted sensitivity analyses through a “leave-one-out” approach to demonstrate that individual SNPs did not unduly influence the causal relationship between exposure and outcome. All statistical analyses for the MR study were conducted using R software (version 4.2.1) and the “TwoSampleMR” R package.

## Results

### The Causative impact of inflammatory cytokines on migraines

Given the limited scope of genetic variation, a constrained number of SNPs, and their relatively modest effect sizes, we conducted a MR analysis employing a lenient p-value threshold of 5 × 10^−6^. By employing these criteria (r^2^ < 0.001, P < 5 × 10^−6^), we collectively identified 347 SNPs associated with 41 cytokines. The F-statistics for the instrumental variables all exceeded 10, signifying the absence of instrumental variable bias in the study results, thus affirming the reliability of our findings. The influence of each SNP locus on migraines was determined through a two-sample Mendelian Randomization analysis (Supplementary data S1).

**Figure 1:**
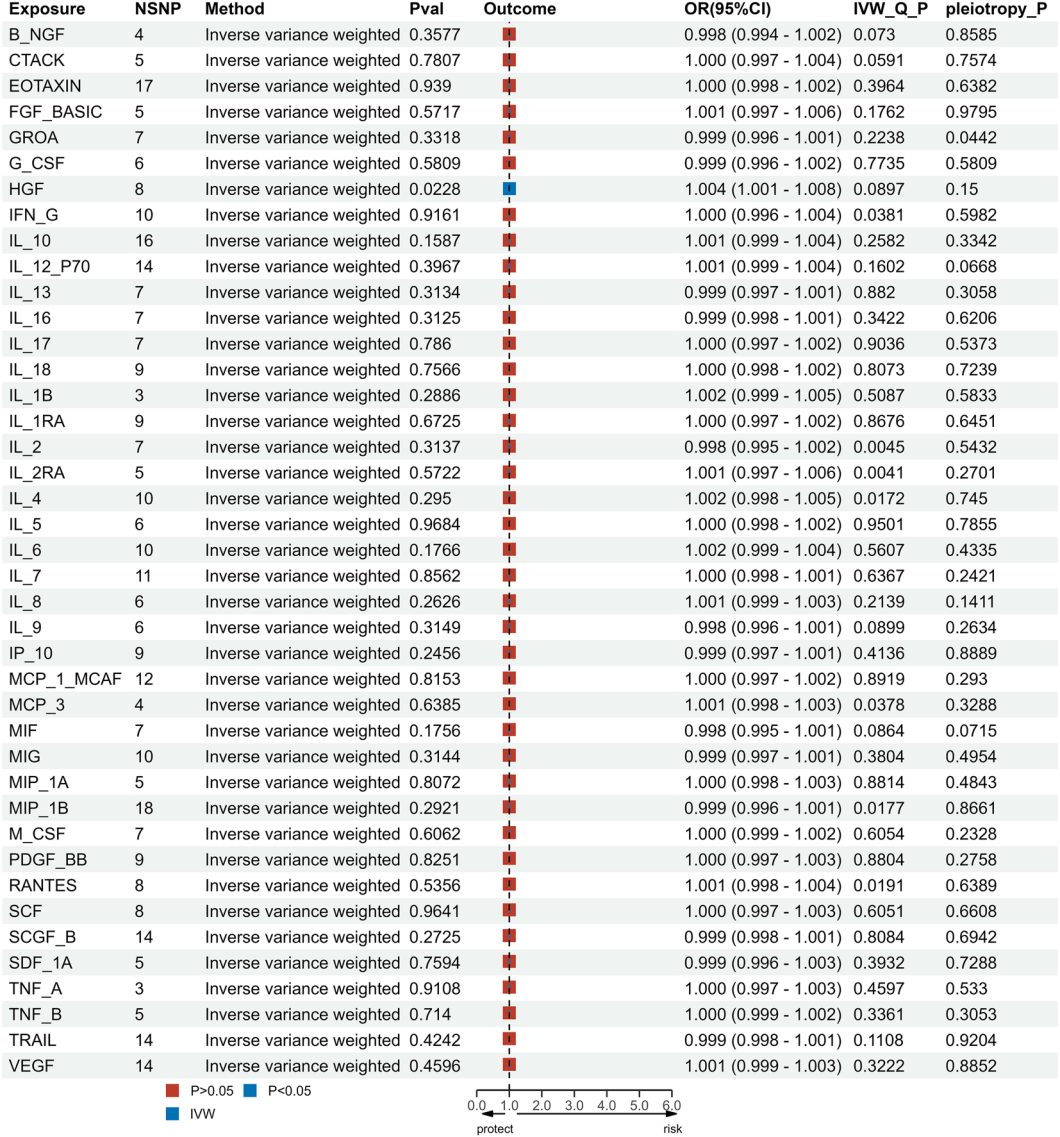
Causal correlations of 41 inflammatory cytokines on migraines. NSNP: the final number of SNPs used in the analysis. OR: the estimated effect of inflammatory cytokines on migraine. IVW_Q_P: the P value of the Cochran Q test. Pleiotropy_P: the P value of the MR-Egger regression intercept hypothesis test.

**Figure 2:**
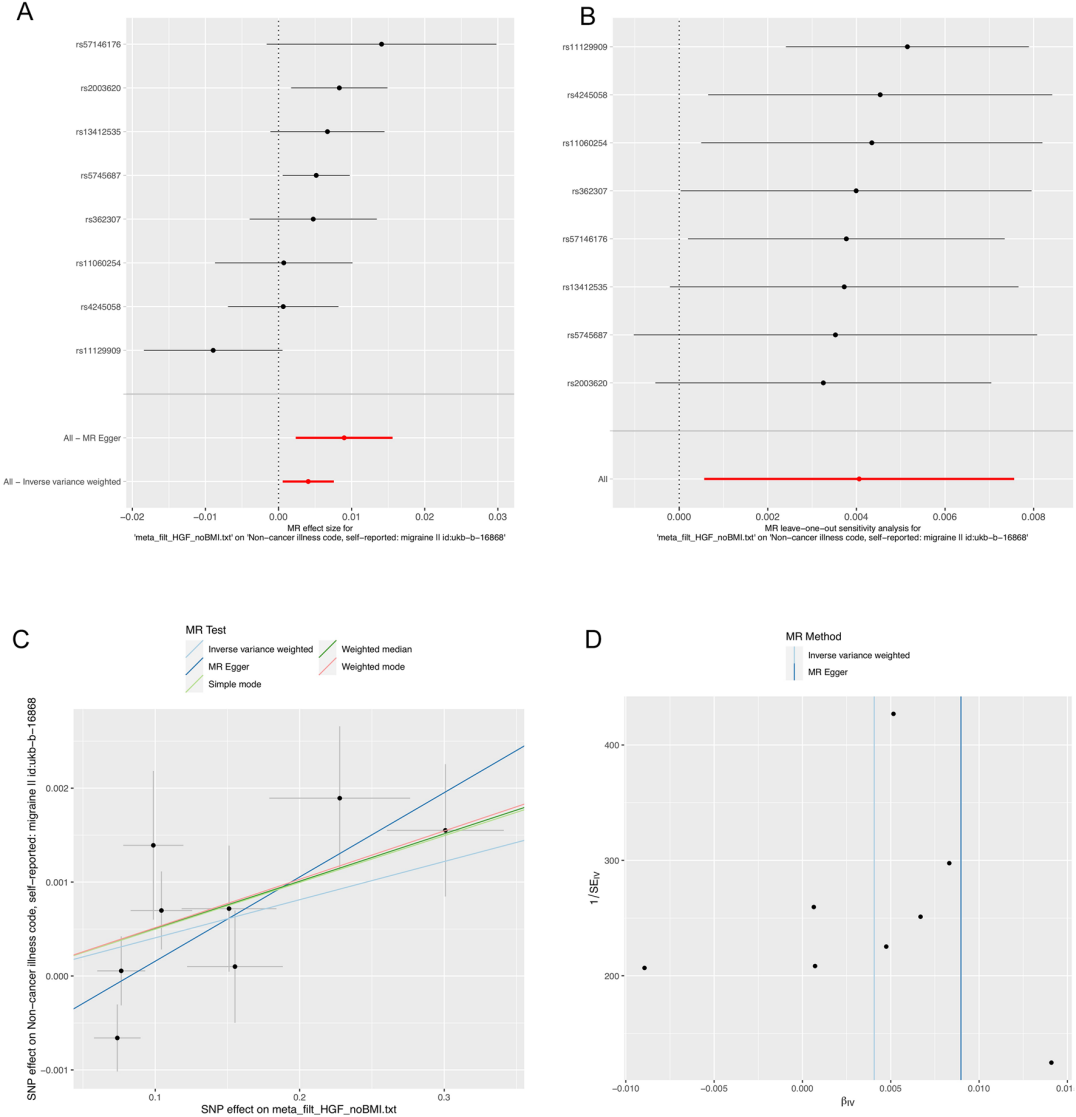
(**A**) Forest plot of the two-samples Mendelian Randomization analysis;Black dots represent the log odds ratio (OR) of migraine with an increase in HGF standard deviation (SD), which is generated using each SNP as an individual instrumental variable.Red dots represent the causal estimates of all SNP combinations using different MR methods. (**B**) Result of “leave-one-out” sensitivity analysis; black dots represent the log odds ratio (OR) of migraine with an increase in HGF standard deviation (SD), which is generated using each SNP as an individual instrumental variable. Red dots represent the causal estimates of all SNP combinations using different MR methods. (**C**) Scatter plot of the two-samples Mendelian Randomization analysis; black dots represent individual SNPs; The slope of the line represents the causal estimate of the MR method. (**D**) Funnel plot of the two-sample Mendelian Randomization analysis; β: the regression coefficient, SE: standard error.

As illustrated in Fig. 1, we screened all 41 inflammatory cytokines using a less stringent threshold (P < 5 × 10^−6^). Among them, HGF displayed a positive association with migraine risk (OR, 1.004; 95% CI: [1.001–1.008]; P = 0.022). Weighted median regression also supported a similar causal relationship (OR, 1.005; 95% CI: [1.001–1.09]; P = 0.007). In the Mr-Egger regression analysis , we found analogous results (OR, 1.009; 95% CI: [1.002–1.016]; P = 0.038) (Figs. 2A, C, 5 and Supplementary data S1). Cochran's Q test in MR Egger and IVW did not reveal significant heterogeneity among the instrumental variables (IVs) in the HGF GWAS (Fig. 1 and Supplementary data S1).To enhance the robustness of these findings, we conducted an Egger pleiotropy test for the included SNP loci, which showed no evidence of horizontal pleiotropy (P > 0.05). The funnel plot similarly indicated the absence of bias in this study (Fig. 2D). Additionally, we performed a leave-one-out sensitivity analysis to assess the influence of each individual SNP locus on the overall causal relationship. Notably, we observed no significant deviations in the observed causal relationship when systematically removing individual SNPs and rerunning the MR analysis (Fig. 2B). This underscores that the estimated effects cannot be attributed to any single genetic instrument. No causal relationship was detected between other inflammatory cytokines and migraines.

### The reverse MR

**Figure 3:**
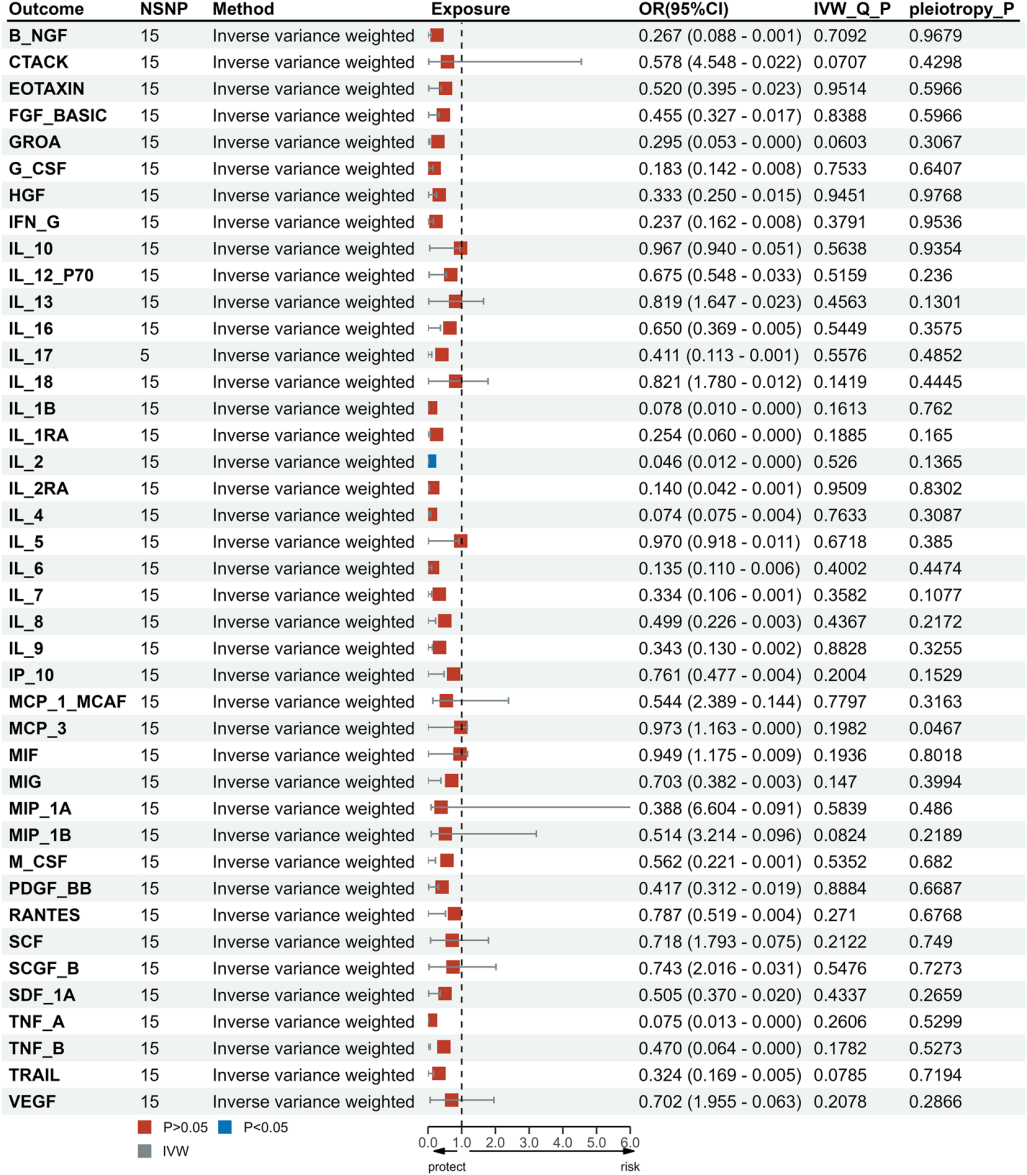
Causal correlations of migraines on 41 inflammatory cytokines. NSNP: the final number of SNPs used in the analysis. OR: the estimated effect of inflammatory cytokines on migraine. IVW_Q_P: the P value of the Cochran Q test. Pleiotropy_P: the P value of the MR-Egger regression intercept hypothesis test.

**Figure 4:**
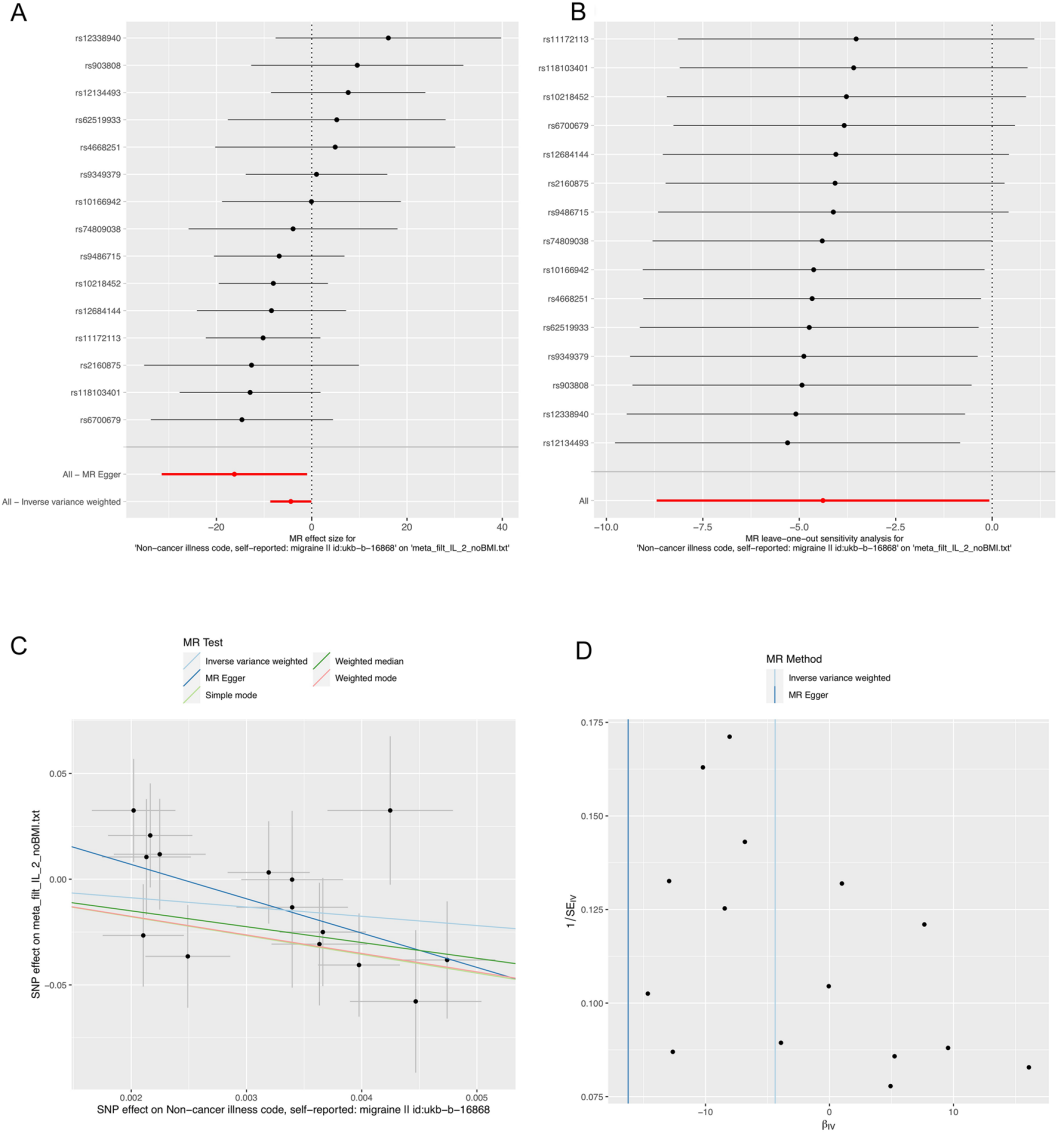
(**A**) Forest plot of the two-samples Mendelian Randomization analysis; black dots represent the log odds ratio (OR) of IL-2 with an increase in migraine standard deviation (SD), which is generated using each SNP as an individual instrumental variable. Red dots represent the causal estimates of all SNP combinations using different MR methods. (**B**) Result of “leave-one-out” sensitivity analysis; black dots represent the log odds ratio (OR) of IL-2 with an increase in migraine standard deviation (SD), which is generated using each SNP as an individual instrumental variable. Red dots represent the causal estimates of all SNP combinations using different MR methods. (**C**) Scatter plot of the two-samples Mendelian Randomization analysis; Black dots represent individual SNPs; The slope of the line represents the causal estimate of the MR method. (**D**) Funnel plot of the two-sample Mendelian Randomization analysis; solid lines represent the effect estimates obtained by different methods.

**Figure 5:**
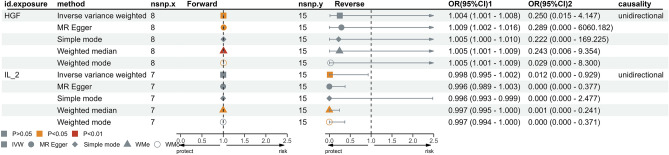
Five methodologies were employed to elucidate the causal relationship between cellular inflammatory factors and migraine.

To explore the possibility of reverse causality, we meticulously screened 15 SNPs that exhibited strong and independent associations with migraines, applying a stringent threshold of P < 5 × 10^−8^. Utilizing the Inverse Variance Weighting (IVW) method, we uncovered a potential link between a heightened risk of migraines and diminished levels of IL-2 (OR, 0.012; 95% CI: [0.000, 0.0929]; P = 0.046). This causal association was consistently corroborated by the Weighted Median analysis (OR, 0.000; 95% CI: [0.000, 0.241]; P = 0.016). However, the MR-Egger method failed to reveal any discernible causal effect (OR, 0.000; 95% CI: [0.000, 0.377]; P = 0.057) (Figs. 3, 4A, C, 5 and Supplementary data S2). To ascertain the robustness of these findings, we conducted an Egger pleiotropy test for the included SNP loci, which yielded no indication of horizontal pleiotropy (P = 0.137). The funnel plot similarly exhibited no signs of bias. Moreover, the adjusted Cochran Q statistics underscored the absence of significant heterogeneity in the effects of the incorporated SNPs (P = 0.526) (Fig. 4D). Furthermore, we undertook a leave-one-out sensitivity analysis to gauge the influence of each individual SNP locus on the overall causal relationship. Impressively, no substantial deviations in the observed causal relationship emerged when systematically eliminating individual SNPs and rerunning the MR analysis (Fig. 4B). This solidifies the notion that the estimated effects cannot be attributed to any single genetic instrument. Importantly, no causal relationship was observed between migraines and other inflammatory cytokines.

## Discussion

In this MR analysis of two samples, we investigated the causal relationships between 41 inflammatory cytokines, including interleukins and chemokines, as exposures and migraine as the outcome. Our results suggest that HGF may be a potential cause of migraine. Additionally, migraine may lead to decreased levels of IL-2 through a causal pathway if it is defined as an exposure in MR. No evidence of reverse causality was found between any single inflammatory cytokine and migraine. Therefore, it can be concluded that HGF may play a dominant role in the early onset of migraine, while IL-2 is more likely to be downstream in the disease progression.

The role of the immune system in migraine attacks is often overlooked. Studies have shown that migraine patients may have an immune dysfunction, as evidenced by abnormal levels of plasma cytokines during and before attacks^17^. People with impaired immune function are more likely to experience migraine symptoms. In addition, sumatriptan treatment can induce changes in the transcription of immune genes in blood after migraine attacks. Studies have shown that migraine patients have higher levels of IL-1β and IL-6, and lower levels of IL-10, compared to healthy controls^18^. A study published in 2021 measured cytokine levels in the blood of migraine patients and healthy controls, and found that TNF-α was elevated, but IL-1β was not compared to controls^19^. Protein measurements in cerebrospinal fluid (CSF) showed that transforming growth factor-β (TGF-β), IL-1β receptor antagonist, and monocyte chemoattractant protein-1 levels were significantly different in migraine patients compared to controls^20^. It is important to note that these measurements were taken within the central nervous system.

HGF, also known as scatter factor, was originally purified from plasma and platelets^21^. It was thought to be a mitosis promoter that stimulates the proliferation of hepatocytes. However, recent studies have found that HGF has a wide range of biological effects, participating in the growth, regeneration, and remodeling of cells in various organs and tissues of the body. It is widely present in the nervous system, and HGF and HGF mRNA are expressed in different regions of the nervous system. HGF is a new neurotrophic factor in the nervous system, with protective and growth-promoting effects on nerve tissue^22^. In the nervous system, it plays an important role in the development, survival, growth, and guidance of neurons, and the nutrition of muscles.HGF is a self-secreted factor that plays a role in the development of sympathetic fibroblasts and neurons. It has been shown to significantly increase the axonal outgrowth of nerve growth factor-dependent sympathetic neurons^23^. This suggests that HGF may cause migraine by affecting the release of neurotransmitters. Additionally, studies have shown that HGF is produced in increased amounts in most organs and tissues when they are damaged^24^. Research has shown that the HGF-Met signaling pathway plays a key role in the development and specialization of nociceptors. Specifically, this pathway appears to be closely associated with the choice of some nociceptors to become peptidergic neurons. Peptidergic neurons are a specific type of sensory neuron that releases peptide neurotransmitters, such as CGRP. These neurons play a critical role in pain transmission and neurotransmitter release. The mechanism of action of HGF in peptidergic differentiation is as follows: When HGF binds to the Met receptor, it activates the Met receptor signaling pathway. The activation of this pathway further inhibits the expression of the Runx1 gene, a key gene involved in neuronal properties and differentiation. Once the expression of Runx1 is inhibited, genes associated with peptidergic differentiation are activated, leading to the peptidergic differentiation of nociceptors. Specifically, the expression of the CGRP gene is activated, leading to the synthesis and secretion of CGRP^25^. In conclusion, although research on HGF in migraine is still in its early stages, our study provides a promising research direction for future studies, which will help to better understand the role of HGF in the pathogenesis of migraine.

IL-2 is a member of the interleukin family that is produced by activated T cells. It binds to IL-2 receptors on the cell surface and stimulates T cell proliferation, development, and differentiation^26^. Different types of T cells have different sensitivities to IL-2, with regulatory T cells (Tregs) being more sensitive than effector T cells (Teffs). This means that low doses of IL-2 can selectively activate Treg signaling pathways without affecting Teffs. This can regulate Treg cell levels and functions, enhance immune suppression, and perform immune regulation^27^.In rodent models of nerve injury and spontaneous experimental autoimmune encephalomyelitis, increasing Treg cell numbers can alleviate mechanical allodynia, while depletion of Treg cells exacerbates mechanical allodynia^28^. Treg cells are known to suppress immune responses and maintain immune homeostasis through a variety of mechanisms, including the production of TGF-β, IL-10, and interleukin-35 (IL-35). These cytokines have all been shown to reduce pain sensitivity in chronic pain models. A recent study showed that low doses of IL-2 can block and reverse pain hypersensitivity in a mouse model of headache^29^. Additionally, Research has have shown that levels of CGRP and vasoactive intestinal peptide (VIP) are significantly higher in the inter-ictal phase of migraine than in the non-migraine state^30^. VIP is a neuropeptide that acts as a neuromodulator and neurotransmitter. It is a potent vasodilator that can regulate smooth muscle activity, epithelial cell secretion, and blood flow in the gastrointestinal tract. VIP is a chemical messenger that is released from nerve terminals and acts locally on cells with receptors to exert neurohormonal and paracrine effects. It is produced by a Th2 lymphocyte population that can promote Th2-type immune responses. In vitro, VIP can inhibit Th1 cells from producing interferon-γ and IL-2 in antigen-activated CD4 T cells^31^.

In summary, we hypothesize that HGF binding to the Met receptor further activates the Met receptor signaling pathway, leading to the peptide-specific differentiation of pain-sensing neurons, promoting the synthesis and release of CGRP. CGRP can also regulate the activity of trigeminal ganglion neurons and glial cells at multiple sites in the trigeminal nervous system by activating CGRP receptors, leading to trigeminal neurovascular reactions. After being stimulated, the trigeminal ganglion and its fibers release vasoactive peptides. These active substances act on the dura mater, causing degranulation of dura mater mast cells and neurogenic inflammation. In addition, VIP acts on Th1 cells on the dura mater to inhibit the production of IL-2. Although there is limited research on HGF and IL-2 in migraine, our study suggests that they play a significant role in migraine pathogenesis. Further studies are warranted to explore these relationships in more detail.

The study has several limitations. First, it was conducted in a single population of European ancestry, so it is not possible to generalize the findings to other populations. Second, the lack of detailed clinical information prevented subgroup analysis, which limited the ability to identify specific causal relationships.

## Conclusion

This MR study identified one upstream regulator and one downstream effector of migraine, which could provide potential therapeutic targets for migraine prevention. However, the associations between inflammatory cytokines and migraine need to be validated in larger cohorts.

### Supplementary Information

Supplementary Information 1.

Supplementary Information 2.

Supplementary Legends.

## Data Availability

All data generated or analysed during this study are included in this published article [and its supplementary information files].
