# Supplementary material for: Exploring the causal relationship between inflammatory cytokines and migraine: a bidirectional, two-sample Mendelian randomization study
Source: Sci Rep. 2023 Nov 8;13:19394. doi: 10.1038/s41598-023-46797-3 (PMC10632361; doi:10.1038/s41598-023-46797-3)
Supplement: Supplementary file 3 — Supplementary Legends. [file 41598_2023_46797_MOESM3_ESM.docx]

**Supplementary data:**

Supplementary data S1. IV-inflammatory cytokines and the forward MR analysis results of inflammatory cytokines and migraine.

Supplementary data S2. IV-migraine and the reverse MR analysis results of inflammatory cytokines and migraine.
